# Atypical centrioles are present in *Tribolium* sperm

**DOI:** 10.1098/rsob.160334

**Published:** 2017-03-15

**Authors:** E. L. Fishman, Kyoung Jo, Andrew Ha, Rachel Royfman, Ashtyn Zinn, Malathi Krishnamurthy, Tomer Avidor-Reiss

**Affiliations:** Department of Biological Sciences, The University of Toledo, Toledo, OH 43607USA

**Keywords:** centriole, *Tribolium*, sperm, Ana1, atypical centrioles, PCL

## Abstract

Typical centrioles are made of microtubules organized in ninefold symmetry. Most animal somatic cells have two centrioles for normal cell division and function. These centrioles originate from the zygote, but because the oocyte does not provide any centrioles, it is surprising that the zygotes of many animals are thought to inherit only one centriole from the sperm. Recently, in the sperm of *Drosophila melanogaster*, we discovered a second centriolar structure, the proximal centriole-like structure (PCL), which functions in the zygote. Whether the sperm of other insects has a second centriolar structure is unknown. Here, we characterized spermiogenesis in the red flour beetle, *Tribolium castaneum*. Electron microscopy suggests that *Tribolium* has one microtubule-based centriole at the tip of the axoneme and a structure similar to the PCL, which lacks microtubules and lies in a cytoplasmic invagination of the nucleus. Immunostaining against the orthologue of the centriole/PCL protein, Ana1, also recognizes two centrioles near the nucleus during spermiogenesis: one that is microtubule-based at the tip of the axoneme, suggesting it is the centriole; and another that is more proximal and appears during early spermiogenesis, suggesting it is the PCL. Together, these findings suggest that *Tribolium* sperm has one microtubule-based centriole and one microtubule-lacking centriole.

## 1. Introduction

The centriole is a cellular organelle that is essential for fertilization and embryo development (reviewed in [1]). Most somatic cells have a pair of centrioles, each consisting of microtubules arranged in ninefold radial symmetry. The older centriole is known as the mother centriole, and it forms the axoneme (a microtubule-based subcellular skeletal structure) of the cilium (aka flagellum) during interphase. The younger centriole, known as the daughter centriole, matures into a mother centriole in preparation for cell division. Before mitosis, the centrioles duplicate (each centriole serves as a platform for the formation of a single new centriole nearby) to have four centrioles, and two are inherited by each daughter cell [2–4]. Since two centrioles are required for normal cell division, a fundamental question in reproductive biology is from where the first two centrioles in a zygote arise [5].

In most sexually reproducing animals, the female gamete does not provide any functional centrioles to the zygote; rather, the male gamete (spermatozoon) provides the centrioles [5,6]. Because the zygote requires two centrioles, the sperm of many animals, like fish, amphibians and nematodes, have two centrioles. These two centrioles are the daughter centriole that is called the proximal centriole, which is found near the nucleus, and the mother centriole that is called the distal centriole, which is found at the base of the sperm tail's axoneme [7–9]. These two sperm centrioles function after fertilization in the zygote and serve as a platform for the formation of new zygotic centrioles. These first two pairs of centrioles are inherited by daughter cells and duplicated to generate the centrioles of the animal's somatic cells. This pattern of centriole inheritance ensures that all somatic cells inherit exactly two centrioles, as abnormalities in number can cause devastating developmental defects and cancer [10]. Since precise centriole number is critical, it is surprising that the insect and mammalian spermatozoa appear to have only one centriole, and the origin of the second centriole is unknown (reviewed in [11]).

In insects, only the centriole that forms the axoneme is present (the homologue of the mammalian distal centriole) [12]. This centriole has been found with a variable number of microtubules, but most commonly have either nine triplet or doublet microtubules [13], as has been shown in *Drosophila melanogaster* (triplets) [14] and *Mastotermes darwiniensis* (doublets) [15]. Alternative microtubule arrangements have been observed in species like *Sciara* (60–90 singlet microtubules) [16] and *Caenorhabditis elegans* (nine singlet microtubules) [8]. Since all these centrioles have microtubules, the structural definition of a centriole is a cylindrical subcellular structure made of microtubules usually in ninefold or radial symmetry [17–19].

Recently, an atypical centriole was discovered in *Drosophila* sperm: the proximal centriole-like structure (PCL), which forms during early spermiogenesis (haploid sperm development). The initiation of PCL formation requires the same set of proteins as are required to initiate centriole formation (i.e. Asl, Plk4, Sas-6), and the PCL is composed of centriole-specific proteins (e.g. Sas-4, Ana1 and Bld10) [20]. The PCL is formed at the proximal end of the centriole, later shifts into the centriolar adjunct (a specialized form of PCM found in insect sperm [21]) and finally resides at the base of the nucleus, after the centriolar adjunct is eliminated from the spermatozoon in a process known as centrosome reduction [20,22]. The PCL lacks microtubules and has a novel structure, consisting of a 100 nm wide electron dense wall with a 20 nm wide central tubule and translucent interstitial material between them [22,23]. After fertilization in the zygote, the inherited PCL forms a centrosome that emanates a microtubule aster. The PCL is essential to form a new daughter centriole, and to form one of the spindle poles during cell division [22,24]. Aspects of the PCL's formation mechanism, composition, and function overlap with those of a centriole, although the PCL structure is distinct from that of a centriole. These findings raise the hypothesis that the PCL is a new type of centriole: an atypical centriole, which is structurally defined as a microtubule-lacking cylindrical subcellular structure, made of centriole-specific proteins. Therefore, *Drosophila* sperm has two centriolar structures, one typical and one atypical, which resolves the origin of the second centriole in this species.

In order to test if the PCL is a *Drosophila* ‘anomaly’ or represents a new type of centriole found in insects, we examined the centrioles of the red flour beetle (*Tribolium)* during spermatogenesis (sperm development). *Tribolium castaneum* (red flour beetle) is a representative species of the largest, most diverse eukaryotic order (Coleoptera), including over 300 000 species [25]. *Tribolium* and *Drosophila* shared a common insect ancestor about 300 million years ago, and if the PCL was present in this ancestor, *Tribolium* would be expected to have a PCL. Therefore, studying *Tribolium* will provide insight into the diversity and generality of centriolar structures in insects.

Our findings suggest that *Tribolium* sperm has two centriolar structures: the first is an axoneme-attached centriole with nine doublet microtubules that was previously described in many insect studies, and the second is a new type of centriole that lacks microtubules and has a unique structure that is similar, but not identical, to the *Drosophila* PCL. Together these results suggest that insects have a spectrum of centriolar structures ranging from the typical centriole with radial triplet, doublet or singlet microtubules to the structurally distinct atypical centriole without microtubules. It also suggests that, while the structure of sperm centrioles can be diverse, the number is conserved.

## 2. Results and discussion

### 2.1. *Tribolium* spermiogenesis

**Figure 1.**
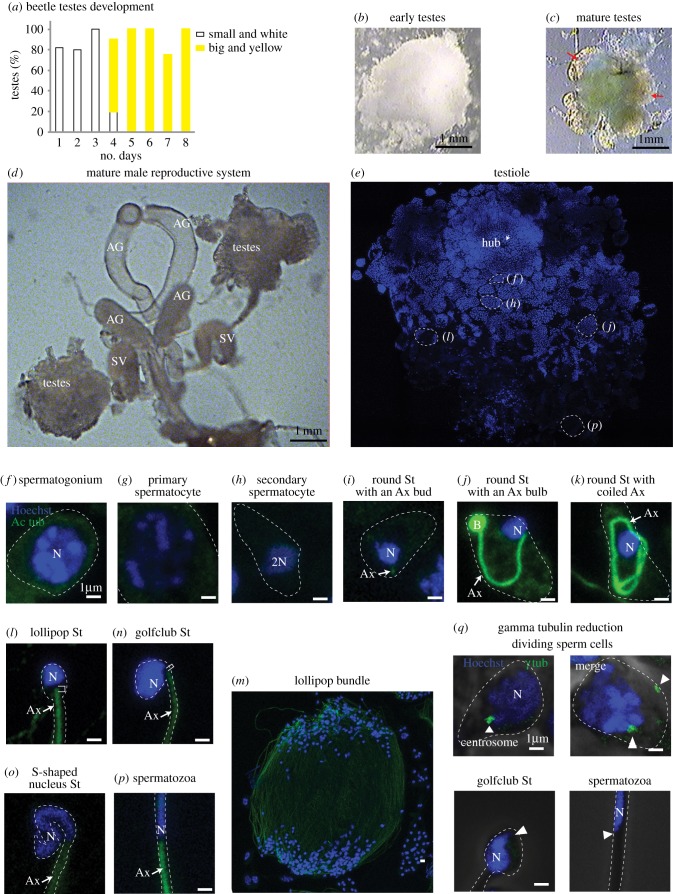
*Tribolium* spermiogenesis. (*a*–*c*) As the beetles eclose from the pupa, their testes change (graph in *a*). They start as small white testes, surrounded by a big white cloud of fat (*b*). Around day 4, they change to large and yellow with no more than six clear lobes (testioles marked with red arrows) and very little fat (*c*). Though the early testes (*b*) appears equal in size to day 4 testes (*c*), in reality, the early testes fat fills the volume difference between the smaller testes and the larger ones on day 4. (*d*) The complete male reproductive system. (*e*) A single testiole stained with Hoechst (blue) to recognize the nuclei. Specific areas are presented in panels (*f*)–(*p*) as labelled. The Hoechst staining becomes lighter in later-stage sperm. (*f*–*p*) Sperm cells at distinct stages are stained with Hoechst (blue) and anti-acetylated-tubulin (green). (*q*) Anti-γ-tubulin (green) staining is observed in dividing-stage sperm but is undetectable in late-stage sperm (golfclub spermatid and spermatozoa) at the expected site of the centriole (arrowhead). Abbreviations: AG, accessory gland; SV, seminal vesicle; Ax, axoneme; N, nucleus; B, bulb; St, spermatid. Scale bars, 1 µm.

*Tribolium*'s spermatogenesis was previously described briefly, but no immunofluorescent systematic study of spermiogenesis has been published [26,27]. Therefore, to study the sperm centrioles, we first characterized *Tribolium*'s male reproductive organ (testis) and later spermiogenesis using immunofluorescence. *Tribolium* testes size grows dramatically after eclosion from pupae. Initially, on day 2, it is small, delicate and encased in white fat (figure 1*a,b*). On day 4, the testes become yellow and large, and have several (at most 6) lobes (testioles) that are arranged around the vas deferens, which carries the spermatozoa to the seminal vesicle [28] (figure 1*c*,*d*). In each testiole, spermatogenesis is organized along an axis that begins with stem cells at the ‘hub’ and ends with spermatozoa at the vas deferens (figure 1*e*).

Next, we characterized *Tribolium* spermiogenesis. The early sperm cells (spermatogonia) are small, round and have round nuclei with uniform Hoechst DNA staining. During cell division, the condensed DNA is stained more intensely (figure 1*f*). The post-mitotic spermatocytes are big, round and have big nuclei that contain patchy Hoechst staining (figure 1*g*). They are organized in a cyst, but meiosis occurs asynchronously [27]. The secondary spermatocytes, which are found between meiosis I and II, are smaller than primary spermatocytes and have more condensed DNA (figure 1*h*). Early round spermatids that are formed after meiosis have an axoneme bud labelled by acetylated tubulin (figure 1*i*). A small gap between the acetylated tubulin-labelled axoneme and the nucleus is maintained throughout spermatogenesis. Later, the axoneme elongates and curves within the elliptical spermatid, while the tip of the axoneme forms a bulb of intensely stained acetylated tubulin (figure 1*j*). Next, the axoneme elongates further and coils around the nucleus (figure 1*k*). After elongation, the spermatids, with round nuclei and long axonemes that appear as lollipops (‘lollipop spermatids’), are organized antiparallel to each other [26] (figure 1*l*,*m*). Then, spermatids transition to ellipsis-shaped nuclei with a severely kinked neck region (‘golfclub spermatids’) (figure 1*n*), and as the spermatids mature, the nuclei narrow and twist into an S-shape (‘S-spermatid’) (figure 1*o*). Lastly, the nuclei straighten in late spermatids, and spermatozoa have a needle shaped nuclei (figure 1*p*).

The early sperm centrosome is labelled by the universal centrosomal marker γ-tubulin, and the marker is later eliminated during spermiogenesis in a process known as centrosome reduction [5]. Anti γ-tubulin immunostaining shows two dots in dividing sperm cells, but not in differentiating spermatids or spermatozoa (figure 1*q*), suggesting that, like in other animal sperm, the *Tribolium* sperm centrosome is reduced during spermiogenesis. Note that in *Drosophila*, proteins are eliminated during centrosome reduction, but the sperm centriole and PCL are maintained with some structural changes in spermatozoa [22].

### 2.2. The *Tribolium* axoneme-attached centriole has doublet microtubules

The centrioles of insect sperm have structural diversity, but with the exception of the PCL, they all have microtubules [12]. To investigate the number, structure and microtubules of centrioles in *Tribolium* sperm, we performed high-pressure freezing–freeze substitution (HPF-FS) and transmission electron microscopy (TEM) serial section analysis at the junction between the nucleus and flagellum. This technique was integral in determining the PCL structure in *Drosophila*, as traditional chemical fixation tends to disrupt its delicate ultrastructure [22,23].

**Figure 2.**
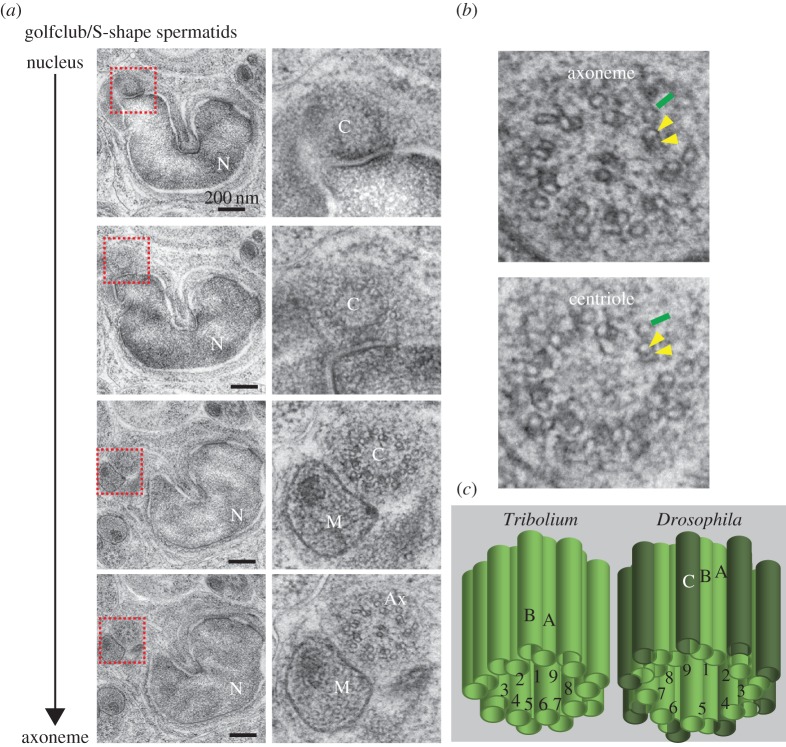
The centriole of *Tribolium* spermatids has doublet microtubules. (*a*) High-pressure freezing (HPF), freeze substitution (FS) and serial section TEM analysis of *Tribolium* golfclub/S-shape spermatid showing the transition from a centriole lacking central microtubules to an axoneme with central microtubules. Abbreviations: Ax, axoneme; C, centriole; M, mitochondria; N, nucleus. (*b*) Magnification of axoneme and centriole cross-sections. Yellow arrowheads: the microtubules in the doublet microtubules; green line: accessory microtubule. (*c*) Model of the axoneme-attached centriole. The centriole is made of doublet microtubules labelled A and B in ninefold symmetry, which is different than *Drosophila*'s sperm's axoneme-attached centriole's triplets, labelled A, B and C.

To study the centriolar structures in *Tribolium* sperm, we selected the intermediate spermatid stage (golfclub/S-shape) because *Drosophila's* PCL is observed most robustly in this stage. We found that in the sperm flagella, the axoneme in the spermatid is composed of nine accessory tubules, with nine doublets (microtubules A and B) and two central microtubules, as previously described [27]. Traditionally, this organization is referred to as a 9 + 9 + 2 axoneme and is found in many insect groups including other beetles [27,29,30] (figure 2). The base of the axoneme is made of nine accessory tubules, with nine doublets and no central microtubules (9 + 9 + 0), suggesting this is the centriole. This doublet microtubule-based centriole arises from the previously described pair of centrioles with triplet microtubules in spermatocytes [27]. This suggests that the axoneme-attached centriole is modified throughout spermatogenesis as part of centrosome reduction, which has been described in other animals [22,31]. Since centrioles can have nine doublets [32,33], we hypothesize that the axoneme-attached structure is the centriole in *Tribolium*.

### 2.3. *Tribolium* sperm has a proximal centriole-like structure

**Figure 3.**
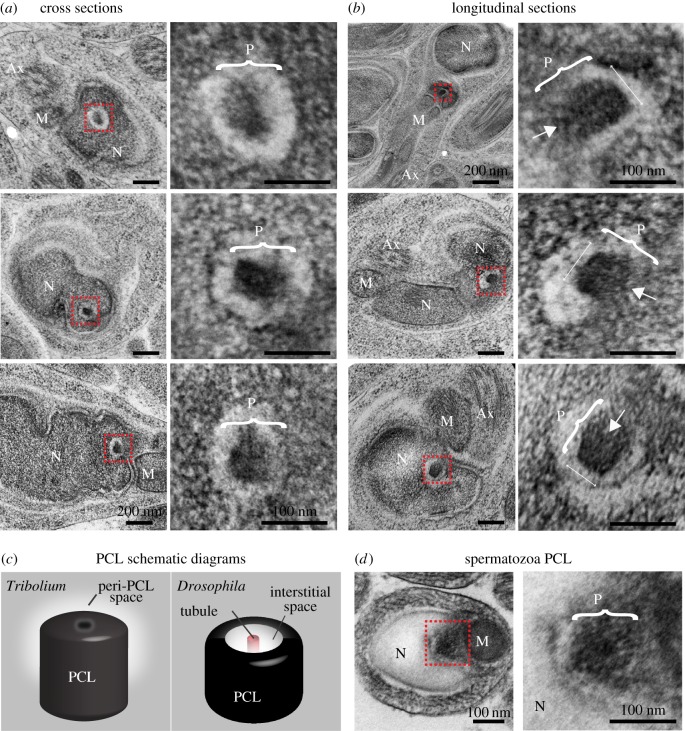
The 2nd centriole of *Tribolium* spermatids lack microtubules. (*a*,*b*) Representative HPF-FS TEM semi cross-section (*a*) and semi-longitudinal (*b*) analysis of *Tribolium* golfclub spermatids. Images on the right are insets of images on the left, signified by the red box. Brackets indicate the axis representing the PCL diameter. Arrows indicate the location of the barely detectable electron-dense central core. (*c*) A model of the second centriolar structure in spermatids. The structure of the PCL (P) is slightly different than *Drosophila*'s PCL. (*d*) Representative TEM sections of the second centriolar structure in *Tribolium* spermatozoa. Abbreviations: Ax, axoneme; M, mitochondria; N, nucleus. Scale bars, 1 µm.

To investigate the presence of a second centriole in the sperm, we analysed the nucleus and axoneme junction. We found a distinct structure between the axoneme-attached centriole and the nucleus (figure 3*a*,*b*). This structure is located in a cytoplasmic invagination of the nucleus and has no microtubules. It is composed of an electron-dense structure with a diameter of 84 ± 6 nm (*n* = 13), and is surrounded by translucent material. The overall shape and the dimensions of this structure are similar to the *Drosophila* PCL (figure 3*c*) [22]. In cross-section, the structure does not possess the central tubule found in the *Drosophila* PCL (figure 3*a*), but a barely detectable electron-dense central core can be observed in longitudinal and semi-longitudinal sections (figure 3*b*). Also, the position differs from that of the *Drosophila* PCL, which is found adjacent to the axoneme-attached centriole. However, the electron-dense structure's position in *Tribolium* is similar to the position of the human proximal centriole: between the base of the nucleus and the axoneme-attached distal centriole [34,35]. This structure is observed in spermatozoa, suggesting it is maintained throughout spermiogenesis (figure 3*d*). Therefore, we hypothesize that this second structure is the homologue of the *Drosophila* PCL. This *Tribolium* PCL lacks microtubules and therefore would traditionally not be defined as a centriole; however, we propose that, like the *Drosophila* PCL, the *Tribolium* PCL is an atypical centriole.

### 2.4. *Tribolium* Ana1 labels two spermatid centrioles

**Figure 4.**
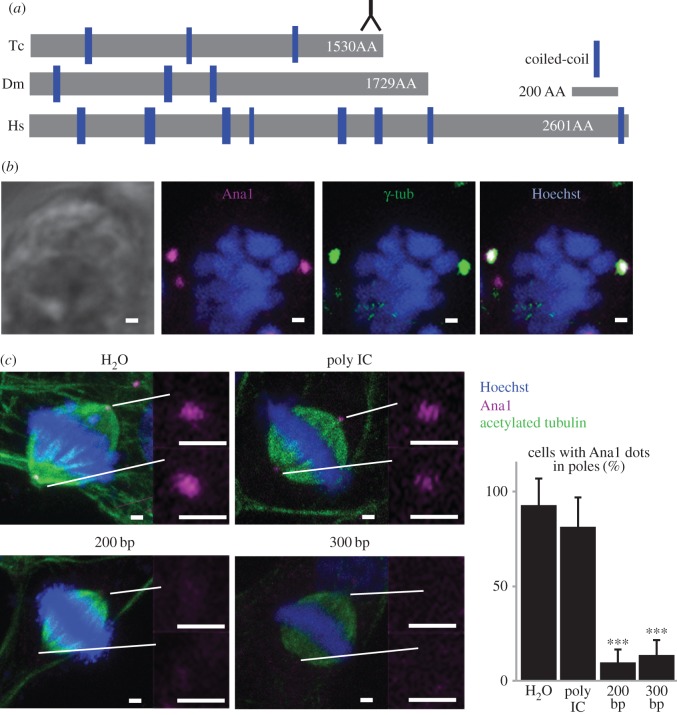
*Tribolium* Ana1. (*a*) Schematic comparison of *Drosophila* (Dm) Ana1, *Tribolium* (Tc) Ana1 and human CEP295 (Hs). Inversed ‘Y’ symbol indicates the region recognized by the antibody. (*b*) Colocalization of γ-tubulin and Ana1 in dividing TcA cells. (*c*) Ana1 labelling is reduced in TcA cells when treated with two different lengths of RNAi fragments against Ana1 (200 and 300 bp), but is not diminished significantly when treated with water (H_2_O) or a non-specific double-stranded RNAi (Poly IC). ***p < 0.001 by *t*-test. *n* = 3. Scale bars, 1 µm.

To test the hypothesis that the *Tribolium* PCL is the second centriolar structure, we tested if it contains the PCL marker and conserved centriolar protein Ana1/CEP295 [20,36,37]. Reciprocal BLASTp between *Drosophila* and *Tribolium* identified a large (172 KD) protein that is 28% identical and has three predicted short coiled-coil domains (figure 4*a*) (hypothetical protein TcasGA2_TC014427) [38]. We generated an antibody against the C-terminus of *Tribolium* Ana1 (amino acids 1506–1530). As expected from a centriolar marker, this antibody recognizes two dots (which often appear as one large, fused dot) that colocalize with γ-tubulin in the spindle poles of dividing *Tribolium* sperm cells and BCIRL-TcA-CLG1 cells (figure 4*b*). RNAi treatment of the BCIRL-TcA-CLG1 cells significantly reduced the Ana1 staining (figure 4*c*), indicating that the antibody is specific to Ana1.

### 2.5. Anti-Ana1 recognizes two centriolar structures in spermatids

**Figure 5.**
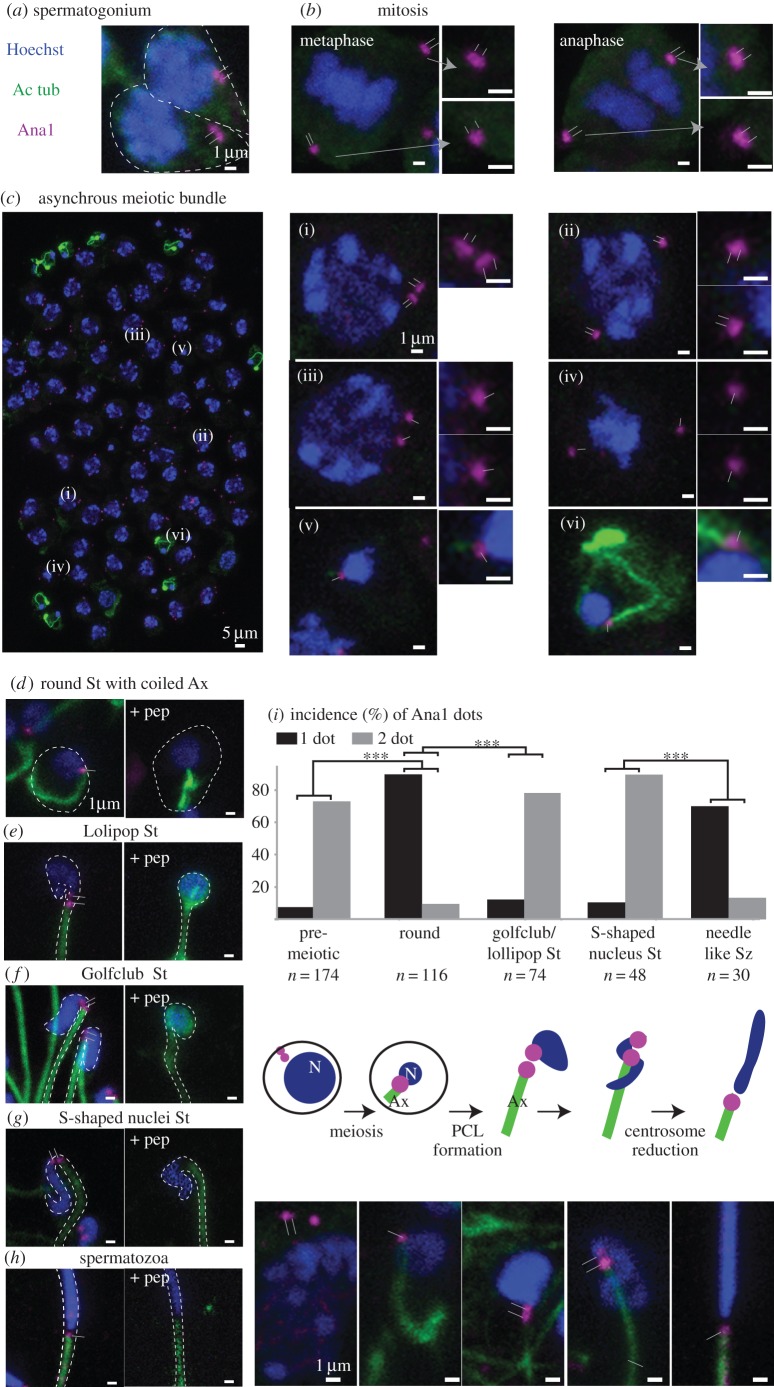
A 2nd centriole forms during *Tribolium* spermatogenesis. (*a*–*h*) Anti-Ana1 labelling throughout *Tribolium* spermatogenesis. (*c*) As expected, an asynchronous meiotic bundle has spermatocytes, each with two centrosomes with a total of four centrioles (i–iii). Each secondary spermatocyte has one centriole at opposite poles (iv), and early spermatid stages have one centriole (v–vi). (*d*–*h*) Anti-Ana1 dots are found at the base of the nucleus throughout spermiogenesis and this staining is abolished in the presence of a competitive peptide. (*i*) Quantification of Ana1 dots in different stages of sperm: one dot (black); two dots (grey). A schematic diagram showing the emergence of the second centriolar structure. *p* < 0.001 by chi-square test. Abbreviations: Ax, axoneme; St, spermatid; Sz, spermatozoa; N, nucleus. Thin lines indicate position of centriole and PCL. Scale bars, 1 µm.

To characterize the centrioles during spermatogenesis, we stained testes with the anti-Ana1 antibody. Interphase spermatogonia have two cytoplasmic Ana1-labelled centrioles that are adjacent to one another (figure 5*a*). Mitotic spermatogonia have two centrioles that localize to each spindle pole (figure 5*b*). Primary spermatocytes have two pairs of cytoplasmic Ana1-labelled centrioles; the centrioles are often very close together so that they appear as one elongated dot (figure 5*c*(i)). During meiosis I, each pair of centrioles migrates to opposite poles (figure 5*c*(ii)). At the end of meiosis I, the secondary spermatocyte has two centrioles (figure 5*c*(iii)). Dividing secondary spermatocytes (figure 5*c*(iv)) produce two round spermatids, with one Ana1-labelled centriole in each cell. This Ana1-labelled centriole is located between the nucleus and the axoneme, and the axoneme is initially shorter and later is elongated (figure 5*c*(v,vi)). This location suggests that the Ana1-labelled axoneme-attached centriole found in round spermatids is homologous to the *Drosophila* centriole and the human distal centriole. Interestingly, in *Tribolium,* the Ana1-labelled axoneme-attached centriole is observed in spermatozoa; this is different from *Drosophila*, where Ana1 cannot be detected from both centrioles due to centrosome reduction [39].

During the transition from a round spermatid to a lollipop spermatid, a second Ana1-labelled dot appears between the axoneme-attached centriole and the nucleus (figure 5*d*,*e*). Both this proximal dot and the axoneme-attached centriole persist in golfclub spermatids and S-shaped spermatids (figure 5*f*,*g*). At the S-shaped spermatid stage, the proximal Ana1 dot is in the expected position for the PCL (near the centriole), but its precise position is distinct. The emergence of the Ana1-labelled PCL post-meiosis is consistent with the timing of the emergence of *Drosophila* PCL. After the S-shaped spermatid stage (figure 5*h*), the Ana1 dot is not detected at the *Tribolium* PCL, which is consistent with the reduction of *Drosophila* PCL. Finally, the Ana1 staining is abolished in the presence of a competitive peptide (figure 5*e–h*). Quantification of these data shows significant changes between one and two dots, which correlates with the known timing of meiosis, centrosome reduction and the PCL's emergence in *Drosophila* (figure 5*i*). Together, this immunostaining study indicates presence of a second centriolar structure during *Tribolium* spermiogenesis, which is found between the axoneme-attached centriole and the nucleus.

**Figure 6.**
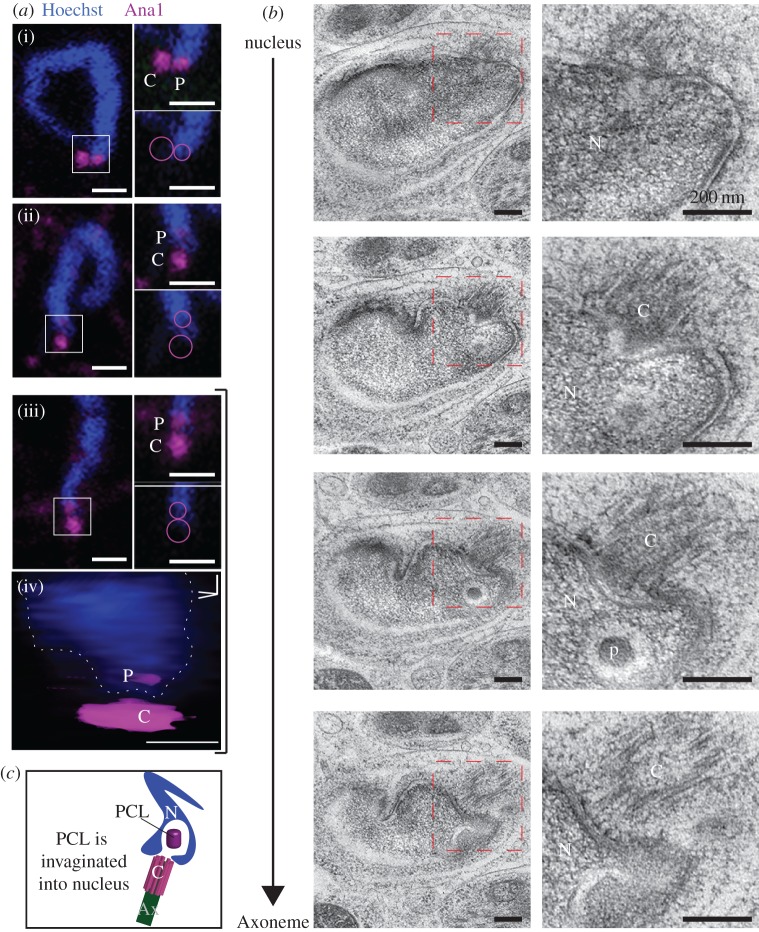
The 2nd Ana-1 labelled centriole is the PCL. (*a*) Single 0.3 µm thick confocal sections showing that the proximal Ana1 dot is located inside the nucleus boundary in golfclub/S-shaped spermatid stage (i–iii). Circles mark the location of the centriole and PCL in the inset. Three-dimensional volume model of the PCL in an S-shaped spermatid iii (iv). Scale bars, 1 µm. (*b*) Serial section TEM analysis showing that only the PCL is found in the cytosolic invagination within the nucleus at the golfclub/S-shape spermatid stage. Scale bars, 200 nm. (*c*) A schematic diagram showing the location of the PCL in a cytosolic invagination within the nucleus. Abbreviations: Ax, axoneme; C, centriole; N, nucleus; P, PCL.

Finally, to investigate if the electron-dense structure we observed using EM is the same as the proximal Ana1 dot, we examined the proximal Ana1 dot and the nucleus base using single frames (figure 6*a*(i–iii)) and three-dimensional reconstitution (figure 6*a*(iv)), and found that the proximal Ana1 dot is located inside the nucleus boundary. Since serial-section electron microscopy finds that the PCL is the only distinct cytosolic structure invaginated to the nucleus base (figure 6*b*,*c*), these data strongly suggest that the proximal Ana1 dot is the *Tribolium* PCL.

## 3. Concluding remarks

Here, we examined the centrioles of *Tribolium* during spermatogenesis and found that *Tribolium* sperm has two centrioles: (i) as described previously, a centriole made of doublet microtubules instead of triplet microtubules, and (ii) a previously undescribed atypical centriole, the *Tribolium* PCL, that is made of a 100 nm electron-dense core surrounded by electron-translucent material. Like *Drosophila*'s PCL, the *Tribolium* PCL localizes near the centriole that forms the axoneme, but its relative position and ultrastructure are distinct. This suggests that insect sperm centrioles can have a range of structures.

There are several potential explanations why the PCL was not detected in the many electron microscopy studies of insect and beetle sperm:

(1) The PCL is small; it is about the size of a single electron microscopy ultrathin section (approx. 70 nm), making it easy to miss.
(2) The PCL is found in different locations relative to the centriole in different species preventing location-based identification.
(3) The PCL has a different structure depending on the species, making it hard to identify based on structural criteria.
(4) The PCL is masked by the electron-dense centriolar adjunct that surrounds it. Electron microscopy studies in a variety of beetles have reported the presence of centriolar adjunct that could be masking the PCL [40–42]. Observing the internal structure of the PCL, which distinguishes it from the centriolar adjunct, requires non-standard electron microscopy techniques, such as high pressure freezing with freeze substitution. Alternatively, immunostaining can be used to distinguish the PCL, which is made of centriolar proteins, from the centriolar adjunct, which is made of PCM proteins.

Centrioles can be defined in many ways based on their structure, protein composition and function [11,17]. Traditionally, centrioles are defined based on their structure and composition; centrioles are defined as cylindrical subcellular structures containing microtubules [17–19]. Our studies suggest that the sperm's atypical centrioles are small (approx. 100 nm) electron-dense subcellular structures composed of centriole-specific proteins, which lacks microtubules.

Recognizing centriolar structures solely based on microtubules may be causing a systemic bias against structurally atypical centrioles. Perhaps atypical sperm centrioles are more common than is currently acknowledged. The diversity of centriole structures may be of particular importance when considering mammalian sperm. While human and primate spermatids begin with two centrioles, spermatozoa is thought to possess only one intact centriole because the other centriole degenerates [43]. However, since the structure of atypical centrioles is small, delicate and variable, maybe mammalian sperm possess a currently undetected centriole. Therefore, our findings argue for directed research on whether other animal sperm (i.e. that of insects and mammals) has an atypical centriole in addition to their typical centriole.

In light of broader knowledge on sperm and embryo centrioles, our findings argue for a conserved model for centriole inheritance during sexual reproduction. In this model, the spermatozoon inherits exactly two centrioles with diverse structures. Altogether, these findings expand the definition of centriolar structures to include those that do not have microtubules, but have centriole-specific proteins.

## 4. Methods

### 4.1. Beetle cultivation and dissection

Vermillion White and Georgia-1 red flour beetles were grown in a 30°C incubator in mason jars with filter paper lids. Beetles were given whole-wheat flour, supplemented with 5% brewers yeast, to eat. Testes were dissected from males by lightly squeezing using forceps at the thorax until their genitalia were exposed. A second pair of forceps was used to hold and pull the genitalia while maintaining firm pressure on the beetle's thorax. Upon removal, testes were selected, isolated from the remainder of the reproductive system and placed in 1X PBS.

### 4.2. Antibodies

Antibodies were generated in rabbits against the C-terminus of *Tribolium castaneum* Ana1 (Cys-RAKDVEQRFFELQDHSGKKE) and affinity purified by Pacific Immunology. This antibody was used at 1 : 800 for immunofluorescence and did not work well for westerns. GTU88 anti-γ-tubulin and acetylated tubulin (Sigma T6557 and T7451 respectively) were used at 1 : 100.

### 4.3. Immunofluorescence

Slides with beetle testes were prepared using a modified version of Basiri *et al*. [44]. Dissected beetle testes were placed in 20 µl of freshly prepared 3.7% formaldehyde (Sigma, 252549) for five minutes on a charged slide (Azer Scientific, EMS200A+). Formaldehyde was then wicked away and replaced with 10 µl of 1X PBS. The testes were then teased apart, and a sigmacote (Sigma, SL2) coverslip was pressed onto the sample. Then, the slide was placed in liquid nitrogen for at least 10 min. The slide was removed quickly from nitrogen, then the coverslip was removed using forceps, and, lastly, the slide was placed in a pre-chilled coplin jar of ice-cold methanol for 2 min. Next, the slide was placed in 1X PBS for 1 min, then placed for 10 min in fresh 1X PBS with 3% Triton X-1000 at room temperature. PBST-B was prepared by adding 1% BSA to PBST, and slides were then placed in PBST-B for 20 min. Antibodies diluted in PBST-B with 1% RNase A were added to the slides and they were covered in parafilm. Next, the slides were placed in a humidity chamber for at least 1 h incubation at room temperature, or overnight incubation at 4°C. Slides were washed three times in PBST for 5 min each. The secondary antibody mixture was prepared by diluting the antibody in PBST with 1% RNase A. Secondary antibodies used were: 1 : 800 Donkey anti-Mouse conjugated to Alexa Fluor 488 (Jackson ImmunoResearch 715-545-150), 1 : 600 Donkey anti-Rabbit conjugated to a Cy5 fluor (Jackson ImmunoResearch 711-165-152), or 1 : 800 Goat anti-Rabbit conjugated to Alexa Fluor 650 (Fisher 84546 respectively), and 2 µg ml^−1^ Hoechst 33258. The secondary antibody mixture was added to slides, and they were covered in parafilm. Next, they were incubated for at least 1 h at room temperature. Lastly, slides were then washed three times with PBST for 5 min each, followed by three times with 1× PBS for 5 min each. Immunostained slides were sealed and imaged using a Leica confocal microscope Sp8.

Sperm images were taken using magnification of 640X and zoom of 6× with 512 × 512 pixel density. Figures 1, 4 and 5 show a projection using Leica LAS X software. Figure 6 shows a single Z section and three-dimensional rendering using Leica LAS X software. Using Photoshop, images were cropped to 200 pixels by 100 pixels, or 100 pixels by 100 pixels (10 × 10 or 25 × 25 for insets), the intensity was modified to allow easy visualization, and the panels were resized to 300 dpi for publication.

To perform peptide inhibition, a mixture of primary antibody and 10 µg ml^−1^ of peptide was incubated at 4°C for 1 h prior to staining.

### 4.4. High-pressure freezing–freeze substitution and transmission electron microscopy

For TEM analysis of *Tribolium*, the testes and seminal vesicles of *Tribolium* were dissected and immediately processed using the high-pressure freezer system (Leica EM HPM100) in 20% BSA. The frozen samples were dehydrated and en bloc stained using a freeze substitution preprocessor (Leica EM AFS2) in 96% acetone with 1.5% OsO4 (osmium crystals were dissolved in acetone and 4% water). The freeze substitution started with −90°C for 6 h, then warmed up from −90°C to −10°C over 15 h (5.3° slope). Then, the samples were warmed to −3°C over 1 h (7° slope) while being washed with 96% acetone (at −7°C). Then, they were warmed from −3°C to 4°C over 1 h (7° slope) while being washed twice with 100% acetone. Lastly, the dehydrated samples were infiltrated and embedded in EMBed 812 resin while warming up to room temperature. The ultrathin sectioning (70 nm) was performed using an ultramicrotome (Leica EM UC6), and sections were post-stained with 6% uranyl acetate (in 1 : 1 70% ethanol and 100% methanol), and Reynolds lead citrate (3–4%) (in pre-boiled ddH2O). Then, the sections were imaged using TEM (JEOL 1400-plus) operating at 80 kV.

### 4.5. Cell culture

TcA cells were acquired from Dr Cindy Goodman at the USDA [45]. They were grown in a 29°C incubator without CO_2_ and with Ex-Cell 420 media (Sigma, 14420), supplemented with 10% FBS and 1% streptomycin.

### 4.6. RNAi

*Tribolium* genomic DNA was purified using chloroform/phenol extractions. Then, we amplified two fragments of Ana1 (200 bp and 300 bp) using Ana1 primers (see below) with a T7 promoter sequence (underlined) added on in both forward and reverse directions. Next, we generated dsRNAi using the T7 flash transcription kit from Epicentre. We added the RNAi to the cell culture media at a concentration of 100 ng ml^−1^. RNAi was incubated for 96 h, and then cells were fixed in ice-cold methanol for 5 min and stained for immunofluorescence.

Ana 1 primers:

**Table:** 

T7-F200 T7-F300 T7-R200 T7-R300	TAATACGACTCACTATAGGGAAACCACAACGGTCAAGACGAAAC TAATACGACTCACTATAGGGACACTTCCGCGAAAAAATGACGATC TAATACGACTCACTATAGGGTTTCTTGGAGCATTTGCACGAATC TAATACGACTCACTATAGGGCCTCTTCAGGGATCACATCGCTGG

### 4.7. Statistical methods

Experiments were repeated at least three times (*n* > 3), and statistical analyses (average ± standard deviation) were done with excel. A two-tailed, unpaired Student's *t*-test was used to determine *p*-value (*p*). Chi-squared tests were done to compare two categorical variables (one focus versus two foci) to acquire a *p*-value. *p*-value designations are: *p < 0.05, **p < 0.01, ***p < 0.001.
